# Assessing stroke severity using electronic health record data: a machine learning approach

**DOI:** 10.1186/s12911-019-1010-x

**Published:** 2020-01-08

**Authors:** Emily Kogan, Kathryn Twyman, Jesse Heap, Dejan Milentijevic, Jennifer H. Lin, Mark Alberts

**Affiliations:** 1 Janssen Research & Development, LLC, Raritan, NJ USA; 2 Janssen Scientific Affairs, LLC, Titusville, NJ USA; 3 Hartford HealthCare, Hartford, CT USA

**Keywords:** Database, Outcomes research, Real-world evidence

## Abstract

**Background:**

Stroke severity is an important predictor of patient outcomes and is commonly measured with the National Institutes of Health Stroke Scale (NIHSS) scores. Because these scores are often recorded as free text in physician reports, structured real-world evidence databases seldom include the severity. The aim of this study was to use machine learning models to impute NIHSS scores for all patients with newly diagnosed stroke from multi-institution electronic health record (EHR) data.

**Methods:**

NIHSS scores available in the Optum© de-identified Integrated Claims-Clinical dataset were extracted from physician notes by applying natural language processing (NLP) methods. The cohort analyzed in the study consists of the 7149 patients with an inpatient or emergency room diagnosis of ischemic stroke, hemorrhagic stroke, or transient ischemic attack and a corresponding NLP-extracted NIHSS score. A subset of these patients (*n* = 1033, 14%) were held out for independent validation of model performance and the remaining patients (*n* = 6116, 86%) were used for training the model. Several machine learning models were evaluated, and parameters optimized using cross-validation on the training set. The model with optimal performance, a random forest model, was ultimately evaluated on the holdout set.

**Results:**

Leveraging machine learning we identified the main factors in electronic health record data for assessing stroke severity, including death within the same month as stroke occurrence, length of hospital stay following stroke occurrence, aphagia/dysphagia diagnosis, hemiplegia diagnosis, and whether a patient was discharged to home or self-care. Comparing the imputed NIHSS scores to the NLP-extracted NIHSS scores on the holdout data set yielded an R^2^ (coefficient of determination) of 0.57, an R (Pearson correlation coefficient) of 0.76, and a root-mean-squared error of 4.5.

**Conclusions:**

Machine learning models built on EHR data can be used to determine proxies for stroke severity. This enables severity to be incorporated in studies of stroke patient outcomes using administrative and EHR databases.

## Background

Stroke is the fifth leading cause of death in the US and a primary focus for improving patient outcomes and healthcare quality [1, 2]. The National Institutes of Health Stroke Scale (NIHSS) is a widely accepted, clinically-validated measurement of stroke severity. The NIHSS score serves as an important guide for clinicians to effectively offer guidance about prognosis and disability associated with acute stroke [1, 3–5].

The NIHSS score is defined as the sum of 15 individually evaluated elements, and ranges from 0 to 42. Stroke severity may be categorized as follows: no stroke symptoms, 0; minor stroke, 1–4; moderate stroke, 5–15; moderate to severe stroke, 16–20; and severe stroke, 21–42 [6, 7]. NIHSS scores are not part of structured data in electronic health records (EHR); rather, stroke severity is recorded as free text in physician notes. The lack of a formal stroke severity assessment in large EHR databases is a limitation of real-world evidence patient outcome studies related to stroke [1, 8]. Therefore, this type of machine learning approach may be useful for quantifying stroke severity given the limited availability of clinically assessed NIHSS scores in real-world evidence databases [9], aiding such practices as payer modeling for case mix risk adjustment or assessing quality outcomes. Using billing codes from administrative claims data of the single-payer, compulsory enrollment healthcare program in Taiwan, Sung and colleagues developed several models to derive a stroke severity index and validate its performance against the NIHSS [9–11]. Developing a machine learning model for stroke severity based on claims or EHR data from the United States presents unique challenges due to the fact that the US healthcare system is a multi-payer and provider system financed and delivered through a combination of private and public resources.

The objective of this study was to retroactively impute NIHSS scores for all patients with newly diagnosed stroke in a multi-institution EHR database by leveraging machine learning techniques. Imputed NIHSS scores will enable large-scale real-world observational studies to incorporate a measure of stroke severity in research studies of disease burden in these patients.

## Methods

### Database

The Optum© de-identified Integrated Claims-Clinical dataset combines adjudicated claims data with EHR data. The EHR database is derived from more than 50 healthcare provider organizations in the United States, including more than 700 hospitals and 7000 clinics that provide care to more than 91 million patients in the United States. Optum©‘s Integrated Claims-EHR dataset is statistically de-identified under the Expert Determination method consistent with Health Insurance Portability and Accountability Act (HIPAA) and managed according to Optum© and customer data use agreements [12, 13]. The Integrated dataset links both claims and clinical data for approximately 14 million matched individuals, and is generated by Optum© using a proprietary algorithm that incorporates both salting and cryptographic hashing. The EHR information contained in the integrated dataset includes medications prescribed and administered, lab results, vital signs, body measurements, diagnoses, procedures, and information derived from physician notes using Natural Language Processing (NLP).

The NIHSS scores are a part of the information derived from the physician notes [14], and these scores were used as an outcome variable when training and evaluating model performance. Because some invalid values were originally extracted from the physician notes (e.g. values which are not integers within NIHSS range), rigorous pre-processing was applied to exclude as many invalid NIHSS scores as possible. This exclusion criterion was defined in collaboration with Optum and evaluation was completed on the remaining extracted NIHSS values to ensure accuracy. When a patient had multiple NIHSS scores during their inpatient stay following stroke, the maximum score was used to capture the overall severity of the stroke. This study incorporated EHR data from January 2007 through September 2016.

### Study population

**Fig. 1:**
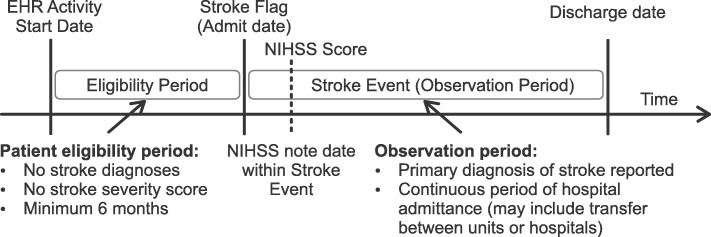
Schematic diagram of study design. Schematic diagram of study design, including timeline and patient inclusion requirements. EHR: electronic health record; NIHSS: National Institutes of Health Stroke Scale

Patients were included in the study if they had a primary diagnosis of stroke (hemorrhagic [the International Classification of Diseases (ICD)-9: 431; ICD-10 I61.XX], ischemic [ICD-9: 433.XX-434.XX, 436; ICD-10: I63.XXX], or transient ischemic attack (TIA) [ICD-9: 435.X; ICD-10: G45.9]) in an inpatient or emergency room setting, which was defined as the stroke event (Fig. 1). Additionally, patients were required to have a real NIHSS score (extracted from physician notes) during the stroke event. Patients were also required to have been in the database for at least 6 months prior to the stroke diagnosis.

From the Optum© de-identified Integrated dataset, 7149 patients were identified who met these inclusion and exclusion criteria. Prior to developing the machine learning model, patients were randomly grouped into a training set (*n* = 6116, 86%) and hold-out test set (*n* = 1033, 14.4%). Model development, parameter tuning, and feature selection were completed using the training dataset.

### Feature engineering and feature selection

Relevant patient demographics (e.g. age, gender) and billing codes related to procedures, diagnoses, prescriptions/medications, hospital visit information, and comorbidities were collected to form the initial set of 8023 potential features. All features were created during the inpatient hospitalization following stroke occurrence (Fig. 1), except the Charlson Comorbidity Index, which was estimated based on data prior to the stroke [15]. Diagnoses codes were from the ninth and tenth revisions of the ICD-9 and ICD-10 [16]. In order to create features for machine learning models that are agnostic to coding version an equivalency mapping provided by The Centers for Medicare & Medicaid Services (CMS) was leveraged. As the granularities of diagnoses codes are different in ICD9 and ICD10 revisions this mapping includes many-to-many relationships. By starting with all diagnosis codes within the stroke events for the patient cohort, and recursively incorporating any diagnosis codes that are equivalent according to the CMS mapping, disjoint diagnosis code groups were created. Binary features were then formed by checking each patient for the presence or absence of any diagnosis within a given ‘diagnosis code group’ during the patients’ stroke event.

Additionally, simple presence/absence features were created for procedures coded with the Current Procedural Terminology (CPT4), Healthcare Common Procedure Coding System – HCPCS procedure codes, Bergenson-Eggers Type of Service codes (BETOS), patient discharge status, diagnosis-related group assigned to the inpatient stay, drug class of prescriptions written, drug class of medications administered, and routes by which medications were administered. Counts of procedures (e.g. CPT4) and BETOS code groups within a patient’s stroke event were also included in the initial feature set. Other features included patient’s age at the time of stroke, gender, and length of hospital stay following stroke occurrence.

**Fig. 2:**
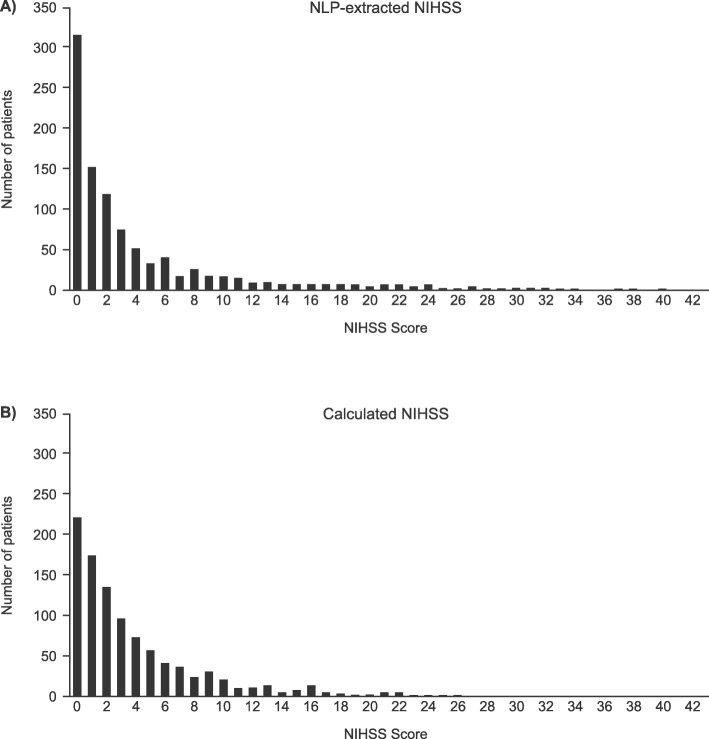
NIHSS Distribution. Distribution of (**a**) real NLP-extracted NIHSS scores and (**b**) model-imputed NIHSS scores for the hold-out test set (*n* = 1033). Model-imputed scores were rounded down to nearest integer

During the feature selection process, features with near zero variance or with high correlation (> 0.9) to another feature were removed. In the latter case, only the feature more highly correlated with the response variable was retained. A response-balanced subset of the training cohort was created for this step in the process, by randomly selecting an equal number of patients from each of 5 stroke severity categories [6] (*n* = 183 per category, *n* = 915 total); this step was necessary such that the feature engineering process was not affected by the skewed distribution of stroke severity categories (i.e., more patients in less severe categories than in more severe categories, Fig. 2). After initial feature selection, the remaining 619 features were used for the subsequent modeling step.

### Machine learning model development

**Fig. 3:**
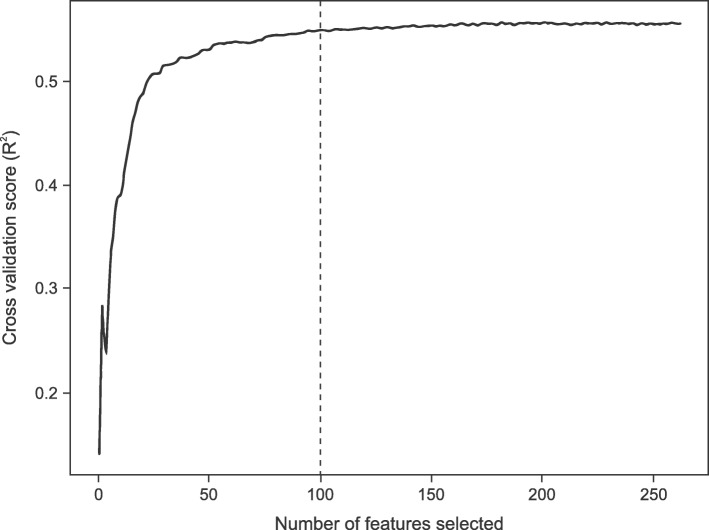
Feature selection based on cross-validated model performance. Feature selection based on cross-validated model performance on the training data. Mean model performance was calculated on 3-fold cross-validated subsets for feature selection. Maximum cross-validated performance was achieved with the top 226 features incorporated into the model; however, minimal gains in performance were seen beyond 100 features

The imputed NIHSS scores were ultimately compared to the real NIHSS scores (extracted from physician notes) in the hold-out test dataset to assess model performance using the coefficient of determination (R^2^), the Pearson correlation coefficient (R), and root-mean-squared error (RMSE). During our initial model development, performance was compared across a set of models developed by several machine learning approaches including a random forest model, gradient boosting model, neural network, and linear regression. The random forest model, which is a meta estimator used to fit several classifying decision trees on various subsamples of the dataset, had the best performance. Model hyperparameters were optimized using a grid search and performance was evaluated using three-fold cross validation within the training data. (Additional file 1: Table S1). Recursive feature elimination performed on the training data reduced the 619 features further, with only the top 100 features included in the final model. The top 100 features were selected because only minor improvements in performance would have been gained for a substantial increase in model complexity with the inclusion of additional features (Fig. 3).

## Results

Table 1 provides demographic data for patients at the time of stroke for each of the two cohorts (training and hold-out test set). Patients in the hold-out test set (*n* = 1033) were only used once to assess the performance of the final optimized random forest model with 100 selected features. This ensured that performance metrics were not biased by over-fitting and that the model for predicting stroke severity scores is generalizable to data not previously included in model development.

**Table 1:** Patient Demographics and Characteristics

Characteristic	Training set (*n* = 6116)	Hold-out test set (*n* = 1033)	Overall population (*n* = 7149)
Demographics			
Age, mean (SD)	66 (14)	67 (14)	66 (14)
Female, n (%)	3196 (52)	568 (55)	3764 (53)
Region			
Northeast, n (%)	464 (8)	84 (8)	548 (8)
Midwest, n (%)	2388 (39)	389 (38)	2777 (39)
South, n (%)	2957 (48)	496 (48)	3453 (48)
West, n (%)	186 (3)	40 (4)	226 (3)
Other/Unknown, n (%)	121 (2)	24 (2)	145 (2)
EHR data			
NIHSS, median (IQR)	2 (6)	2 (6)	2 (6)
LOS, median (IQR)	3 (5)	2 (4)	3 (5)
Type of stroke^a^			
Ischemic, n (%)	4328 (70.8)	710 (68.7)	5038 (70.5)
Hemorrhagic, n (%)	605 (10.0)	113 (10.9)	718 (10.0)
TIA, n (%)	2235 (36.5)	384 (37.2)	2619 (36.6)
Charlson Comorbidity Index^b^, median (IQR)	1 (3)	1 (3)	1 (3)

*SD* standard deviation, *EHR* Electronic Health Record, *NIHSS* National Institutes of Health Stroke Scale, *IQR* interquartile range, *LOS* length of stay, *TIA* transient ischemic attack

^a^Based on ICD diagnosis codes during stroke event; more than one type may be coded per patient stroke event

^b^Calculated based on patients’ diagnosis codes prior to stroke [15]

**Fig. 4:**
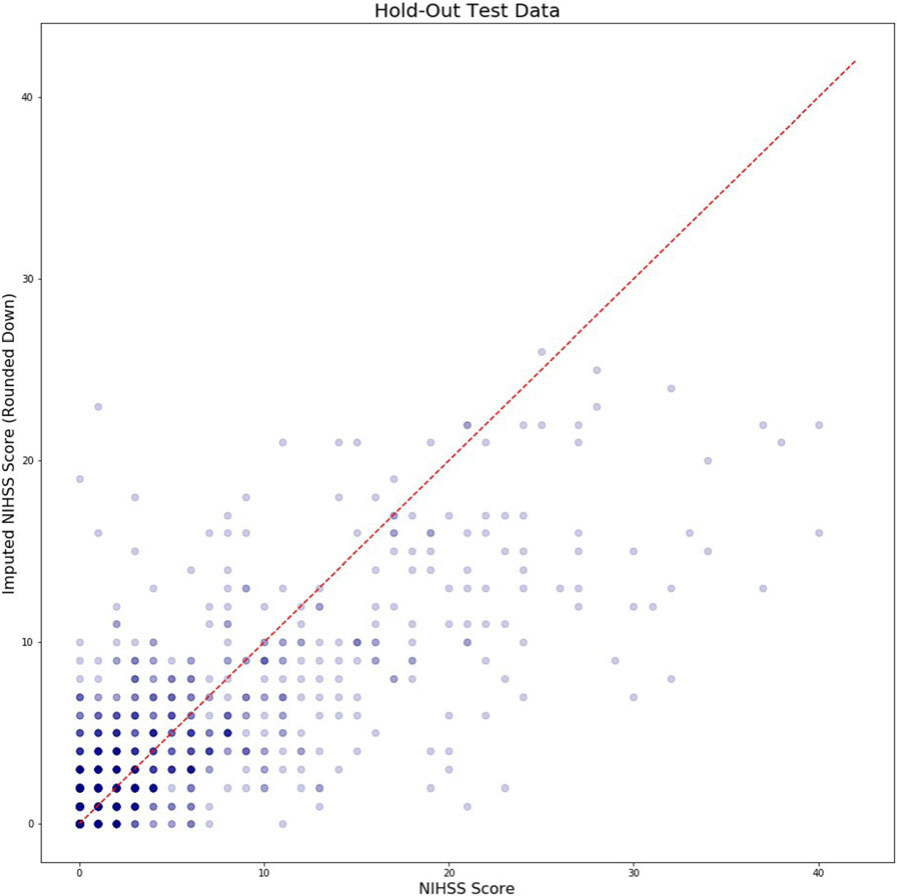
Imputed versus actual NIHSS scores. Imputed versus actual NIHSS scores in the hold-out test cohort. Lighter colored points represent single patients whereas darker points represent multiple overlapping patients

The random forest model achieved an R^2^ of 0.57, an R of 0.76, and a RMSE of 4.5. Figure 4 presents imputed versus actual NIHSS scores. The median (interquartile range, IQR) NIHSS score in the hold-out test cohort was 2 (6) for both the real and imputed NIHSS scores. The distribution of the real NIHSS scores and imputed NIHSS scores for the hold-out test cohort are shown in Fig. 2.

A detailed list of the 100 features included in the final model is shown in Additional file 1: Table S2, ranked in order of relative importance. Top features included death within the same month as stroke occurrence, length of hospital stay following stroke occurrence, aphagia/dysphagia diagnosis, hemiplegia diagnosis, and whether a patient was discharged to home or self-care.

## Discussion

The results of this study demonstrate that machine learning algorithms built on patient treatment and demographic information can be used to determine proxies for NIHSS scores in a real-world evidence database. This is a novel advancement in the ability to quantify stroke severity in large population-based cohorts. This methodology also has the advantage of obtaining these values without the significant cost and time investment required for retrospective chart review studies and enables assignment of a proxy NIHSS value even if the assessment was not completed or documented at the time of stroke occurrence. Applying this model to patients with stroke in the Optum© de-identified Integrated dataset who did not have a real NIHSS score increased the number of patients with stroke with a measure of stroke severity by 6-fold, thus significantly increasing the potential cohort size for any follow-on real-world evidence studies.

Although other methods have been used to determine proxy measures of stroke severity using clinical features available in EHR and claims databases, these methods (such as linear regression) often assume there are linear relationships between the features and stroke severity, which is not always the case [11]. For example, the length of a hospital stay varies based on stroke severity: patients who suffer a moderately severe stroke tend to have a longer length of hospital stay compared to those with low severity strokes who recover more quickly. However, patients with severe stroke have high mortality rates within the first few days of admittance for their strokes and therefore tend to have shorter hospital stays compared to patients with moderately severe stroke. Machine learning algorithms can identify non-linear relationships between features and stroke severity and can incorporate the complex relationships and interactions between features such as the clinical diagnoses relevant to stroke outcomes, treatments including medications and procedures administered to patients at stroke onset, as well as the medical history of patients prior to stroke diagnosis. Machine learning methods are thus well suited to the task of assessing stroke severity based on clinically available information from EHRs.

The distribution of imputed NIHSS scores calculated using this machine learning model as consistent with observations from the population-based Greater Cincinnati/Northern Kentucky Stroke Study [17]. The Cincinnati study determined NIHSS scores from a retrospective chart abstraction of 2233 ischemic stroke cases identified during a 12-month period, with median (IQR) NIHSS values of 3 (6) [17]. This is similar to the results obtained using the machine learning model, where the median (IQR) was 2 (6), and both studies showed a skewed distribution toward less severe strokes. The slightly lower median in the machine learning study may be caused by the inclusion of all types of stroke, including TIAs which tend to be much less severe compared to ischemic stroke. These results contrast with randomized clinical trials in which the enrolled patient population is often selected to include patients with more severe stroke. For example, the Albumin in Acute Stroke study required patients to have an ischemic stroke and baseline NIHSS score of 6 or higher [18] and the Desmoteplase in Acute Ischemic Stroke3 study required patients to have an ischemic stroke and NIHSS score of 4–24 [19].

Documentation of NIHSS scores was evaluated as part of the Get With The Guidelines – Stroke [20]. Over the 10-year study period from 2003 to 2012, the documentation rate was 56.1%, with a median NIHSS score of 4 (IQR, 2–9) and mean of 6.7 (SD, 7.4) [20]. Characteristics associated with NIHSS documentation were those related to eligibility for thrombolysis (e.g. arrival by ambulance and within 3 h of symptom onset) [20]. A modest selection bias was observed reflecting the tendency of hospitals with lower documentation rates to selectively report higher NIHSS scores. The ability to impute NIHSS score with machine learning algorithms may eliminate incomplete documentation issues.

The machine learning model described in this study was developed on a US-based data source and achieved similar performance to models developed from the more uniform, single payer, compulsory healthcare data from Taiwan [9–11]. The R was 0.76 for this US based study and ranged between 0.68 and 0.73 for different models developed in the Taiwan-based study. In the Taiwanese study, NIHSS scores were assessed on admission and recorded directly in the national stroke registry, patients were primarily managed by neurologists, and the features were based on medical billing codes rather than the diagnosis and procedure codes, as these are considered more accurate in Taiwanese health databases due to Taiwan’s universal coverage for hospitalizations and reimbursement system. In contrast, for this study using the US-based data source, real NIHSS scores were extracted using NLP from free text physician notes, attending physician specialty varied, and diagnostic and procedural coding can vary in the multi-provider US healthcare system. Using a more complex machine learning algorithm with significantly more features enabled the US-based model to achieve similar performance to the Taiwan-based model and demonstrates the ability of machine learning methods to handle the systematic differences from diverse EHR systems across various US providers.

This study is subject to several limitations worthy of consideration. Although the current EHR database has captured comprehensive information on diagnoses, administration of treatments and procedures during stroke occurrence, other information which could be critical for model performance including imaging of brain scans was not available. As with all studies based on real-world data, there is the potential for missing records. In addition, healthcare information in the database was not available until January 2007, which precluded the study from capturing information in patients who might have stroke-related diagnosis prior to the year 2007. As such, the first observed stroke occurrence in the data could be a mix of 1st and possibly later stroke diagnosis. Moreover, generalizability of the model to another database remains unclear, as the current model was trained and validated only within a single database. As such, future work is planned to validate the current model in a different EHR database.

## Conclusions

Applying this machine learning method to assess patient’s stroke severity in real-world databases where NIHSS scores are not available enables large scale health-economics and long-term patient outcome studies to incorporate stroke severity. However, in any such endeavor, it would be important to ensure the removal of all features related to any outcomes being studied (and model performance reassessed after removal) to avoid artificially elevated associations. These enhanced studies can potentially accelerate the development of better clinical management and improve patient quality of care [3, 8]. This study represents a novel advanced analytics application to real-world data that could significantly impact drug development and patient outcomes.

## Supplementary information

**Additional file 1: Table S1.** Random Forest Hyperparameters. The parameters of the final model which were obtained through hyperparameter optimization are presented here. **Table S2.** List of Final Model Features. Here, features are ranked by the expected fraction of the samples they contribute to as a measure of feature importance.

## Data Availability

The datasets generated and/or analyzed during the current study are not publicly available because the data source is owned by a third-party (Optum© Integrated Claims-Clinical dataset). Janssen Pharmaceuticals has a license for analysis of data from this source. As such, the authors cannot provide the raw data themselves. Other researchers could access the data by purchase through Optum©.

## Abbreviations

BETOS: Bergenson-Eggers Type of Service codes
CAFR: Chinese Atrial Fibrillation Registry
CMS: Centers for Medicare & Medicaid Services
CPT4: Current Procedural Terminology
EHR: Electronic health records
HCPCS: Healthcare Common Procedure Coding System
HIPAA: Health Insurance Portability and Accountability Act
ICD: International Classification of Diseases
IQR: Interquartile Range
NIHSS: National Institutes of Health Stroke Scale
NLP: Natural Language Processing
R: Pearson Correlation Coefficient
R^2^: Coefficient of Determination
RMSE: Root-mean-squared error
TIA: Transient Ischemic Attack
