# Supplementary material for: Assessing stroke severity using electronic health record data: a machine learning approach
Source: BMC Med Inform Decis Mak. 2020 Jan 8;20:8. doi: 10.1186/s12911-019-1010-x (PMC6950922; doi:10.1186/s12911-019-1010-x)
Supplement: Supplementary file 1 — Additional file 1: Table S1. Random Forest Hyperparameters. The parameters of the final model which were obtained through hyperparameter optimization are presented here. Table S2. List of Final Model Features. Here, features are ranked by the expected fraction of the samples they contribute to as a measure of feature importance. [file 12911_2019_1010_MOESM1_ESM.docx]

**Table S1. Random Forest Hyperparameters.**

The following are the parameters in the final model, which were obtained through hyperparameter optimization.

| **Hyperparameter** | **Value** | **Description** |
| --- | --- | --- |
| Criterion | Mean squared error | Function used to measure the quality of a split |
| Maximum depth | 15 | The maximum depth a tree may grow to |
| Maximum features | All features | The number of features that are taken into account when looking for the best split |
| Minimum samples leaf | 3 | The minimum number of samples required to be a leaf node |
| Number of estimators | 120 | The number of individual regressor trees |

**Table S2. List of Final Model Features.**

Features are ranked by the expected fraction of the samples they contribute to as a measure of feature importance.

| Feature Description | Additional Details/Codes Included |
| --- | --- |
| Date of death within the same calendar month of hospital discharge for stroke event |  |
| Patient length of hospital stay | From the start of the first visit to the end of the last visit in a continuous series of visits. Recorded in days |
| Aphagia/dysphagia diagnosis | ICD9: 78720, V416; ICD10: R130, R1310 |
| Discharged to home or self-care |  |
| Unspecified side hemiplegia diagnosis | ICD9: 34280, 34290; ICD10: G8190 |
| Tissue plasminogen activator administered to patient (based on medications administered, procedural codes, and diagnosis codes) | If a patient had any of the following: medications administered with NDC: 24477004001, 50242004164, 50242004413, 50242008527, 50242012001, 67286004002, ICD Procedural Code: ICD9 99.10, ICD10 3E03017, 3E03317, 3E04317, 3E05317, 3E06317; HCPCS procedural code: J2997, Diagnosis codes: ICD9 V4588, ICD10: Z9282 |
| Aphasia diagnosis | ICD9: 7843; ICD10: R4701 |
| Age of patient at the time of stroke | Calculated based on year of birth |
| Count of procedural codes that fall within M2B Berenson-Eggers Type of Service Codes |  |
| Medications administered by feeding tube |  |
| Patient's Charlson Comorbidity Index prior to stroke | See reference [1] |
| Count of custom procedure codes: Fall risk |  |
| Transient ischemic attack diagnosis | ICD9: 435, 4350, 4353, 4358, 4359; ICD10: G45.9 |
| Count of procedural codes that fall within M2C Berenson-Eggers Type of Service Codes |  |
| Medications administered within the medication class 'Salicylates' |  |
| Narcotic and agonist analgesic combinations medications administered |  |
| Other discharge disposition |  |
| Count of procedure codes: Subsequent hospital care, per day, for the evaluation and management of a patient, which requires at least 2 of these 3 key components: A detailed interval history; A detailed examination; Medical decision making of high complexity. | CPT4: 99233 |
| Count of procedural codes that fall within T1A Berenson-Eggers Type of Service Codes |  |
| Count of procedural codes that fall within M2A Berenson-Eggers Type of Service Codes |  |
| Neurological neglect syndrome diagnosis | ICD9: 7818, ICD10: R414 |
| Medications administered within the medication class 'Gastrointestinal anticholinergics' |  |
| Presence of procedure code: Critical care, evaluation and management of the critically ill or critically injured patient; first 30-74 minutes | CPT4: 99291 |
| Count of procedure codes: Therapeutic procedure, 1 or more areas, each 15 minutes; gait training (includes stair climbing) | CPT4: 97116 |
| Count of procedural codes that fall within O1E Berenson-Eggers Type of Service Codes |  |
| Presence of procedure code: Radiologic examination, abdomen; single anteroposterior view | CPT4: 74000 |
| Count of procedure codes: Radiologic examination, chest; single view, frontal | CPT4: 71010 |
| Count of procedural codes that fall within I2A Berenson-Eggers Type of Service Codes |  |
| Count of procedural codes that fall within T1H Berenson-Eggers Type of Service Codes |  |
| Count of procedural codes that fall within T2A Berenson-Eggers Type of Service Codes |  |
| Count of procedure codes: Respiratory ventilation procedure | ICD9: 9670, 9671, 9672; ICD10: 5A1935Z, 5A1945Z, 5A1955Z |
| Do not resuscitate status diagnosis | ICD9: V4986; ICD10: Z66 |
| Encounter for palliative care diagnosis | ICD9: V667, ICD10: Z515 |
| Count of procedural codes that fall within P6C Berenson-Eggers Type of Service Codes |  |
| Presence of procedure code: Dilation of artery or extirpation of matter from artery | ICD10: 0270346, 027034Z, 02703DZ, 02703ZZ, 02C03ZZ, 037G3ZZ, 037K3DZ, 037L34Z, 037L3DZ, 037N34Z, 03C63ZZ, 03CG3ZZ, 03CG4ZZ, 03CH0ZZ, 03CH3ZZ, 03CJ0ZZ, 03CJ3ZZ, 03CK0ZZ, 03CK3ZZ, 03CL3ZZ, 03CL4ZZ, 03CM0ZZ, 03CP3ZZ, 03CQ3ZZ, 03CY3ZZ, 04703ZZ, 047C3ZZ, 047H3ZZ, 047K3ZZ, 047M3ZZ, 04C00ZZ, 04CC0ZZ, 04CL0ZZ, 04CM3ZZ, 027134Z, 02713DZ, 027234Z, 05C83ZZ, 05CC3ZZ, 05CF3ZZ, 04HY32Z ICD9: 0040, 3812, 0044, 3974, 0045, 0066, 3607, 3950, 0041, 3606, 0061, 0062, 0063, 1755, 0046, 3818, 3949, 0042, 0047, 3803, 3808 |
| Count of procedural codes that fall within T2D Berenson-Eggers Type of Service Codes |  |
| Beta-adrenergic antagonists (beta blockers) w/o ISA medications administered |  |
| Facial weakness diagnosis | ICD9: 78194, ICD10: R29810 |
| Atrial fibrillation diagnosis | ICD9: 42731; ICD10: 480, 482, 4891 |
| Patient gender |  |
| Count of procedural codes that fall within M3 Berenson-Eggers Type of Service Codes |  |
| Medications administered by rectal route |  |
| Cerebral edema/intracranial injury diagnosis | ICD9: 3485, 85400, 85401; ICD10: S061X0A, S06300A, S06890A, S069X0A |
| Count of procedural codes that fall within I2C Berenson-Eggers Type of Service Codes |  |
| Insertion of feeding device | ICD9: 4311; ICD10: 0DH63UZ, 0DH64UZ |
| Count of procedure codes: Initial hospital care, per day, for the evaluation and management of a patient, which requires these 3 key components: A comprehensive history; a comprehensive examination; and medical decision making of high complexity | CPT4: 99223 |
| Count of procedural codes that fall within Z2 Berenson-Eggers Type of Service Codes |  |
| Count of procedure codes: Critical care, evaluation and management of the critically ill or critically injured patient; each additional 30 minutes | CPT4: 99292 |
| Count of procedural codes that fall within T1E Berenson-Eggers Type of Service Codes |  |
| Count of procedural codes that fall within I3C Berenson-Eggers Type of Service Codes |  |
| Dominant-side hemiplegia diagnosis | ICD9: 34281, 34291; ICD10: G8191, G8192 |
| Count of procedural codes that fall within M5D Berenson-Eggers Type of Service Codes |  |
| Count of procedure codes: Echocardiography, transthoracic, real-time with image documentation (2D), includes M-mode recording, when performed, complete, with spectral Doppler echocardiography, and with color flow Doppler echocardiography | CPT4: 93306 |
| Presence of procedure codes: Respiratory ventilation procedure | ICD9: 9670, 9671, 9672; ICD10: 5A1935Z, 5A1945Z, 5A1955Z |
| Count of procedure codes: Treatment of swallowing dysfunction and/or oral function for feeding | CPT4: 92526 |
| Anxiolytic; benzodiazepine medication administered |  |
| Count of procedure codes: Computed tomography, head or brain; without contrast material | CPT4: 70450 |
| Aphasia due to cerebrovascular disease diagnosis | ICD9: 43811; ICD10: I69020, I69120, I69220, I69320, I69820, I69920 |
| Alpha- & beta-adrenergic antagonists medication administered |  |
| Count of procedure codes: Electrocardiogram, routine ECG with at least 12 leads; interpretation and report only | CPT4: 93010 |
| Discharged or transferred to a skilled nursing facility |  |
| Count of procedure codes: Subsequent hospital care, per day, for the evaluation and management of a patient, which requires at least 2 of these 3 key components: A problem focused interval history; a problem focused examination; medical decision making that is straightforward or of low complexity | CPT4: 99231 |
| Count of procedure codes: Hospital discharge day management; more than 30 minutes | CPT4: 99239 |
| Count of procedure codes: Blood count; complete (CBC), automated (Hgb, Hct, RBC, WBC and platelet count) and automated differential WBC count | CPT4: 85025 |
| Altered mental state diagnosis | ICD9: 78097, ICD10: R410, R4182 |
| Other or unspecified hyperlipidemia diagnosis | ICD9: 2724; ICD10: E784, E785 |
| Irritant or stimulant laxative medication administered |  |
| Medications administered topically |  |
| Histamine H2 antagonist medications administered |  |
| Count of procedural codes that fall within I4B Berenson-Eggers Type of Service Codes |  |
| Count of procedural codes: Injection, enoxaparin sodium, 10 mg | HCPCS: J1650 |
| Count of procedural codes: Prothrombin time | CPT4: 85610 |
| Antiemetic agents (5-hydroxytryptamine-3 (5HT-3) antagonist) medication administered |  |
| Count of procedure codes: Blood count; complete (CBC), automated (Hgb, Hct, RBC, WBC and platelet count) | CPT4: 85027 |
| Narcotic agonist analgesics medications administered |  |
| Dysarthria diagnosis | ICD9: 78451; ICD10: R471 |
| Count of procedure codes: Glucose, blood by glucose monitoring device(s) cleared by the FDA specifically for home use | CPT4: 82962 |
| Count of procedure codes: Therapeutic activities, direct (one-on-one) patient contact (use of dynamic activities to improve functional performance), each 15 minutes | CPT4: 97530 |
| Medications administered by other route | Excludes: Feeding tube, Implant, In vitro, Inhalation, Intra-arterial, Intramuscular, Intravenous, Irrigation, Nasal, Ophthalmic, Oral, Other injection, Otic, Rectal, Subcutaneous, Topical, Urinary tract, Vaginal |
| Count of procedure codes: Computed tomographic angiography, neck, with contrast material(s), including non-contrast images, if performed, and image postprocessing | CPT4: 70498 |
| Count of procedural codes that fall within I2C Berenson-Eggers Type of Service Codes |  |
| Insertion of endotracheal tube | ICD9: 9604; ICD10: 0BH17EZ, 0BH18EZ |
| Count of procedure codes: Troponin, quantitative | CPT4: 84484 |
| Count of procedure codes: Magnetic resonance (e.g., proton) imaging, brain (including brain stem); without contrast material | CPT4: 70551 |
| Count of procedural codes: Infusion, normal saline solution, 1000 mL | HCPCS: J7030 |
| Count of procedure code: Cerebral perfusion analysis using computed tomography with contrast administration, including post-processing of parametric maps with determination of cerebral blood flow, cerebral blood volume, and mean transit time | CPT4: 0042T |
| Dementia/delirium diagnosis | ICD9: 2900, 29010,29012, 29013, 29020, 29021, 2903, 2909, 2930, 2931, 29420; ICD10: F0390, F05 |
| Count of custom procedure codes: Pneumo vaccine |  |
| Musculoskeletal symptoms diagnosis | ICD9: 71966, 72989, 78199; ICD10: R296, R29818, R29898, R2990, R2991 |
| Antidepressant medication administered |  |
| Heparins or glycosaminoglycans medications administered |  |
| Count of procedural codes that fall within I1E Berenson-Eggers Type of Service Codes |  |
| Presence of procedure code: Cerebral perfusion analysis using computed tomography with contrast administration, including post-processing of parametric maps with determination of cerebral blood flow, cerebral blood volume, and mean transit time | CPT4: 0042T |
| Count of procedural codes that fall within I1F Berenson-Eggers Type of Service Codes |  |
| Respiratory failure diagnosis | ICD9: 51851, 51881; ICD10: J95821, J9600, J9601, J9602, J9690, J9691, J9692 |
| Speech disturbances diagnosis | ICD9: 78459; ICD10: R4702, R4781, R4789, R479 |
| Non-dominant side hemiplegia diagnosis | ICD9: 34282, 34292; ICD10: G8193, G8194 |
| Antipsychotic medication administered |  |
| Count of procedural codes that fall within P1G Berenson-Eggers Type of Service Codes |  |
| Pneumonitis due to inhaling food/vomit diagnosis | ICD9: 5070; ICD10: J690 |

**Reference**

1. Quan H, Sundararajan V, Halfon P, Fong A, Burnand B, Luthi JC *et al*. Coding algorithms for defining comorbidities in ICD-9-CM and ICD-10 administrative data. Med Care. 2005;43(11):1130-9.
